# Synergistically Enabling Fast‐Cycling and High‐Yield Atmospheric Water Harvesting with Plasma‐Treated Magnetic Flower‐Like Porous Carbons

**DOI:** 10.1002/advs.202204840

**Published:** 2022-11-24

**Authors:** Yifeng Ying, Guifang Yang, Yingle Tao, Qiannan Wu, Haiqing Li

**Affiliations:** ^1^ State Key Laboratory of Materials‐Oriented Chemical Engineering College of Chemical Engineering Nanjing Tech University Nanjing 211816 P. R. China

**Keywords:** atmospheric water harvesting, carbon, magnetic induction heating, magnetic materials, plasma

## Abstract

Sorption‐based atmospheric water harvesting (AWH) offers a promising solution to the water scarcity in arid regions. However, majority of the existing AWH sorbents are suffering from rather poor water productivity due to their slow water adsorption–desorption cycling capability especially when they are applied in high packing thickness. Herein, an oxygen plasma‐treated magnetic flower‐like porous carbon (P‐MFPC) with large open surfaces, abundant surface oxygen‐containing moieties, and excellent localized magnetic induction heating (LMIH) capacity is developed. These merits, together with the use of air‐blowing‐assisted water adsorption and LMIH‐driven water desorption strategy, synergistically allow P‐MFPC with 2 cm of packing thickness to complete a AWH cycling in 20 min and deliver a record 4.5 L_H2O_ kg^−1^ day^−1^ of water productivity at 30% relative humidity. Synergistically enabling such an ultrafast AWH cycling at high sorbent packing thickness provides a promising way for the scalable high‐yield AWH with compact AWH systems.

## Graphical abstract

An oxygen plasma‐treated magnetic flower‐like carbon with rapid atmospheric water adsorption and localized magnetic induction heating‐driven water desorption capability enables ultrafast cycling and high‐yield atmospheric water harvesting in arid regions.

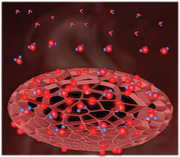

## 1 Introduction

Over half of the global population especially for those living in the arid regions are facing increasingly severe water scarcity.^[^ ^1^ ^]^ Given that the atmosphere holds ≈13 trillion tons of water at any given time in the form of vapor and droplets, atmospheric water harvesting (AWH) has been identified as a promising alternative to alleviate the global water shortage stress.^[^ ^2^, ^3^, ^4^ ^]^ Fog harvesting and dew collection represent the most sophisticated AWH technologies, while their implements are greatly limited by either climate and geographic conditions, or intensive energy consumption concerns.^[^ ^5^, ^6^ ^]^ In contrast, sorption‐based AWH with microporous sorbents has attracted particular interest due to their excellent atmospheric water uptake capacities at low relative humidity (RH), wide range of geographic applicability, and low energy consumption for sorbent regenerations.^[^ ^7^, ^8^, ^9^ ^]^

A sorption‐based AWH mainly involves the use of sorbents to extract water from ambient air and a subsequent heat‐driven water desorption process. To achieve a high‐yield AWH, one of the foremost requirements is to enable a fast water adsorption–desorption cycling of the sorbents, which usually requires the sorbents to have rapid atmospheric water adsorption and desorption kinetics.^[^ ^10^ ^]^ Metal–organic frameworks (MOFs) are the most intensively explored sorbents suitable for AWH in arid environments.^[^ ^11^ ^]^ Unfortunately, the majority of them are suffering from rather slow atmospheric water adsorption kinetics due to the restricted diffusion of water molecules in their microporous matrices.^[^ ^12^, ^13^ ^]^ Compared with MOFs, porous carbons are among the most widely utilized sorbents for gas adsorption and separation due to their outstanding hydrolytic and thermal stability, high surface areas, and porosities,^[^ ^14^ ^]^ while they have attracted much less interest in AWH application due to their intrinsic lack of surface affinity to water molecules. Recent studies indicated that MOF‐derived microporous carbons were able to capture water vapor at low RH with rapid kinetics, which was mainly attributed from the existence of the hydrophilic heteroatoms derived from the MOF precursors.^[^ ^10^ ^]^ Nevertheless, AWH sorbents including such porous carbons and MOFs exhibited desirable water uptake dynamics only if they were utilized in the form of rather thin packing layers (<5 mm).^[^ ^10^, ^12^, ^15^, ^16^, ^17^ ^]^ Increase in the packing thickness of the implemented sorbents would cause a dramatic drop in their atmospheric water adsorption kinetics,^[^ ^12^, ^18^ ^]^ rendering significant limitations in the scale‐up water production with a compact AWH device.

Different from the atmospheric water adsorption process, the highly efficient heat‐driven water desorption could be achieved by the rational choice of the sorbent heating methodologies. Given the natural abundance of solar energy in arid areas, direct solar heating and photovoltaic module‐powered hot‐surface heating have been commonly used to drive the water desorption from sorbents.^[^ ^19^, ^20^, ^21^, ^22^ ^]^ In comparison, the latter heating strategy is more favorable for the high‐yield water production as it allows to proceed multiple water adsorption‐desorption cycling per day regardless of the availability of sufficient sunlight. However, given the intrinsic poor thermal conductivity of the porous sorbents,^[^ ^23^, ^24^ ^]^ the use of such conventional hot‐surface heating and direct solar heating made it rather challenging to realize the sufficient heating and highly efficient water desorption of the sorbents especially when they are utilized in the form of relatively thicker sorbent layers.^[^ ^15^, ^25^ ^]^ This challenge has become one of the major barriers to hinder the high‐yield water production of AWH and the development of compact AWH devices. Distinctly, we have discovered that the use of localized magnetic induction heating (LMIH) delivered by the magnetic nanoparticles embedded within sorbents could lead to a rapid and uniform heating of the entire sorbents, thereby realizing their highly efficient and low‐energy regeneration.^[^ ^26^, ^27^, ^28^ ^]^ As the magnetic field has excellent penetrating capability toward sorbents,^[^ ^26^ ^]^ LMIH‐triggered sorbent regeneration method is particularly ideal for triggering the water desorption from AWH sorbents at large scales.

**Figure 1:**
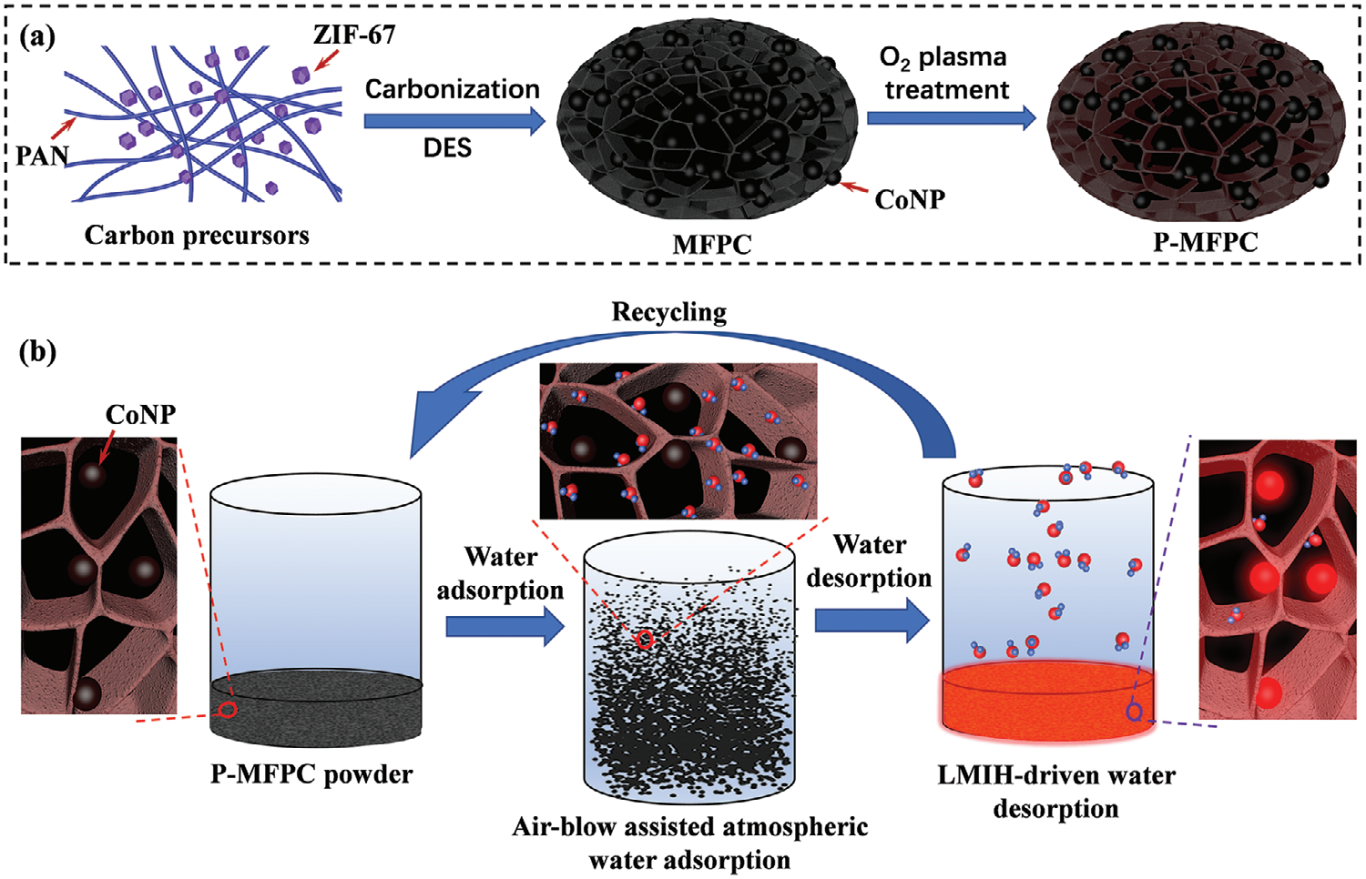
Schematic illustration for the synthesis of the P‐MFPC containing abundant surface oxygen‐containing moieties and CoNPs a) and the use of P‐MFPC for rapid‐cycling AWH: air‐blowing‐assisted dynamic atmospheric water adsorption and LMIH‐triggered water desorption b).

To enable fast water adsorption‐desorption cycling and high‐yield water production of the sorption‐based AWH, we herein developed a plasma‐treated magnetic flower‐like porous carbon (P‐MFPC) containing magnetic cobalt nanoparticles (CoNPs) and abundant surface oxygen‐containing moieties by co‐carbonization of polyacrylonitrile (PAN) and ZIF‐67 in a urea‐zinc chloride deep eutectic solvent (DES), followed by an oxygen plasma treatment (**Figure** **1**a). Such unique structure and composition imparted the resulting P‐MFPC with advantageous features toward fast‐cycling AWH: 1) the flower‐like microstructure made the P‐MFPC have large open surfaces for the air rapid access and exposure, thereby accelerating the atmospheric water adsorption; 2) the abundant oxygen‐containing surface moieties endowed the P‐MFPC with high surface affinity toward water, further promoting its atmospheric water adsorption at low RH; 3) P‐MFPC presented in the form of light powder which could be easily blew up with a fan‐forced airflow. Assisted with such an air‐blowing process, the atmospheric water adsorption of the P‐MFPC could be dramatically accelerated; 4) CoNPs embedded in the matrix of P‐MFPCs were able to rapidly deliver enormous LMIH upon their exposure to an alternating magnetic field, thereby realizing the sufficient heating of the entire P‐MFPCs and triggering the adsorbed water in the P‐MFPC to be highly efficiently released. By synergistically taking advantage of these merits, impressively, the P‐MFPC exhibited extremely fast atmospheric water adsorption and LMIH‐driven water desorption kinetics (Figure 1b) even it was implemented in a high packing thickness (2 cm), thereby enabling a compact P‐MFPC‐based AWH device to complete a water adsorption‐desorption cycle in 20 min and deliver a record 4.5 L_H2O_ kg^−1^ day^−1^ of water productivity at 30% RH and 25 °C.

## 2 Results and Discussion

**Figure 2:**
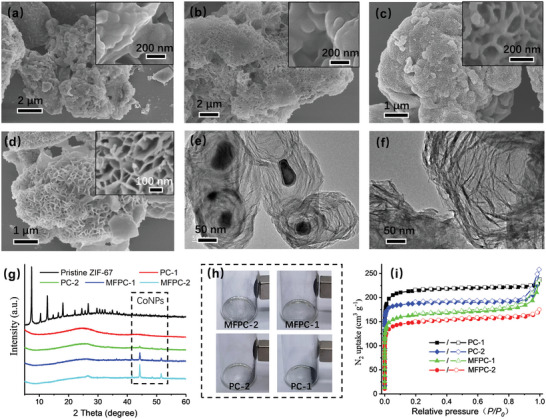
Scanning electron microscopy (SEM) images of PC‐1 a), PC‐2 b), MFPC‐1 c) and MFPC‐2 d), TEM images of MFPC‐2 e) and PC‐1 f), XRD patterns of the pristine ZIF‐67 and the synthesized porous carbons g), digital photographs of the synthesized porous carbons attracted with an external magnet h), and N_2_ adsorption isotherms of the synthesized porous carbons at −196 °C i).

To synthesize P‐MFPC, the pristine MFPCs were firstly synthesized by dispersing a controlled amount of ZIF‐67 MOF crystals (Figure S1, Supporting Information) into a PAN suspension (20 wt%) in urea‐ZnCl_2_ (molar ratio 7:1) DES, followed by a carbonization process (detailed in the experimental section of Supporting Information) based on the previously reported method.^[^ ^29^ ^]^ DECs, consisting of coordinated hydrogen bond donors and acceptors, represent a new type of ionic liquids and have been widely employed in diverse areas due to their low cost, environmental benign, and biodegradability.^[^ ^30^ ^]^ Since the urea‐ZnCl_2_ DES could form continuous hydrogen networks with PAN and result in a highly porous PAN‐derived carbon doped with abundant oxygen that facilitated water adsorption,^[^ ^29^ ^]^ PAN was utilized as the major carbonaceous precursor of MFPCs. Along with the carbonization of PAN, ZIF‐67 not only served as a co‐carbonaceous precursor to produce a hydrophilic porous carbon that was favorable for water adsorption,^[^ ^31^ ^]^ but also provided cobalt metal source (metal centers of ZIF‐67) for their reduction into magnetic CoNPs,^[^ ^32^ ^]^ endowing the resulting MFPC with LMIH capability. With 0 wt% (PAN alone) and 2.8 wt% of ZIF‐67 content in the carbonization reaction systems were utilized, featureless porous carbons (denoted as PC‐1 and PC‐2, **Figure** **2**a,b) were obtained. Interestingly, further increasing the using content of ZIF‐67 to 5.9 wt% and 10.1 wt% resulted in the formation of porous carbons with flower‐like microstructures (denoted as MFPC‐1 and MFPC‐2, Figure 2c,d). In comparison, MFPC‐2 exhibited more defined flower‐like morphology consisting of numerous interfused nanosheets with 30.3 nm of average thickness (Figure 2d). In contrast, using ZIF‐67 alone as carbonaceous precursor only led to the production of featureless magnetic porous carbon consisting of numerous aggregated nanoparticles (Figure S2, Supporting Information). Transmission electron microscopy (TEM) observations indicated that there existed irregular CoNPs randomly distributed within the carbon matrix of the MFPC‐2 (Figure 2e), while no CoNPs were visualized in the PAN alone‐derived PC‐1 (Figure 2f).

The presence of CoNPs in the synthesized carbon materials was further confirmed by X‐ray diffraction (XRD) analysis. As shown in Figure 2g, all the synthesized carbon materials demonstrated the typical broad diffraction peaks of the amorphous carbon in the range of 10^o^–35^o^ whereas in the absence of any diffraction peaks of pristine ZIF‐67 (Figure 2g). In addition, PC‐2, MFPC‐1, and MFPC‐2 exhibited characteristic XRD peaks of CoNPs at 44.3^o^ ((111) lattice face) and 51.6^o^ ((200) lattice face),^[^ ^33^ ^]^ and showed strong attraction toward a magnet (Figure 2h). In contrast, no diffraction peaks of CoNPs and magnetic responsive feature were observed in PAN alone‐derived PC‐1 (Figure 2g,h), further evidencing that CoNPs presented in the PC‐2, MFPC‐1 and MFPC‐2 were derived from ZIF‐67 precursor. All the synthesized carbon materials demonstrated the type I N_2_ adsorption isotherms at ‐196 °C, representing their microporous features (Figure 2i). The Brunauer–Emmett–Teller (BET) surface areas of the PC‐2, MFPC‐1 and MFPC‐2 were 767.3, 638.6, and 582.9 m^2^ g^−1^, which were lower than PAN alone‐derived PC‐1 (874.1 m^2^ g^−1^) due to the presence of nonporous CoNPs and the ZIF‐67 alone‐derived carbon material that had rather lower surface area (251.4 m^2^ g^−1^, Figure S2, Supporting Information).

**Figure 3:**
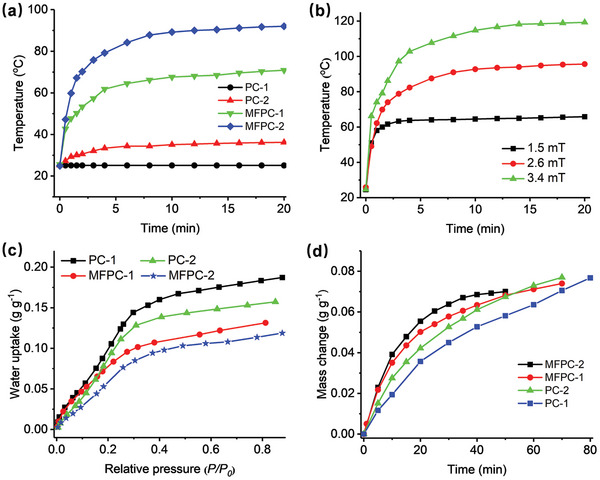
LMIH profiles of the synthesized porous carbons with a 2.6 mT of alternating magnetic field applied a), LMIH profiles of MFPC‐2 with different magnetic field applied b), water vapor adsorption isotherms of the synthesized porous carbons at 25 °C c), the practical atmospheric water adsorption profiles of the synthesized porous carbons at 30% RH and 25 °C d).

The LMIH performance of the synthesized porous carbons was investigated by exposing them to a 2.6 mT of alternating magnetic field, followed by monitoring their surface temperature with an infrared (IR) camera. As shown in **Figure** **3**a, PC‐2, MFPC‐1 and MFPC‐2 could be heated up to 34.4, 66.2, and 88.9 °C in 8 min, respectively, while no heating effect was observed on PC‐1 due to its absence of CoNPs. With variation in the applied magnetic field strength to 1.5 and 3.4 mT, the temperature of the MFPC‐2 could be respectively regulated to 63.9 and 112.2 °C in 8 min (Figure 3b). In comparison, MFPC‐2 showed the best and well‐tunable LMIH capacity. As determined by the gas sorption analyzer measurements at 25 °C, the synthesized porous carbons exhibited the water vapor uptake capacity in the order of PC‐1 > PC‐2 > MFPC‐1 > MFPC‐2 (Figure 3c), corresponding to 0.15, 0.13, 0.10, and 0.085 g g‐1 of water adsorption at 0.3 of *P/P_0_*, respectively. However, under the practical air environment at 30% RH and 25 °C, they demonstrated atmospheric water uptake kinetics in the opposite order of PC‐1 < PC‐2 < MFPC‐1 < MFPC‐2 (Figure 3d). The MFPC‐2 showed the fastest atmospheric water adsorption and took ≈40 min to reach the nearly water saturation at 0.068 g g^−1^, 1.3 times higher than the PC‐1 (0.052 g g^−1^). Such a fast atmospheric water adsorption of the MFPC‐2 was mainly attributed to its flower‐like microstructure which endowed the MFPC‐2 with large open surfaces for facilitating the rapid air access.

**Figure 4:**
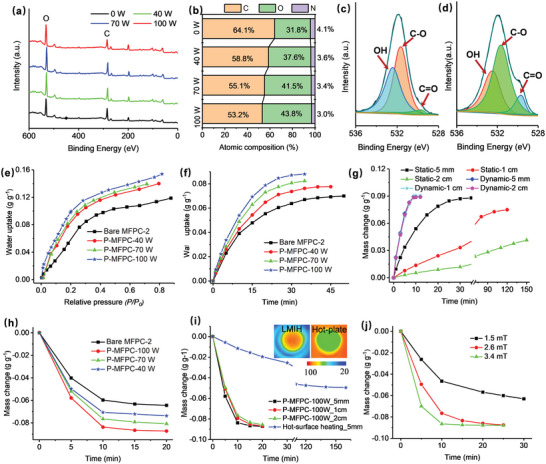
Wide‐scan XPS spectra a) and the corresponding surface atomic composition b) of the bare MFPC‐2 before and after surface treatment with different power of oxygen‐plasma, the high resolution O1s spectra of the bare MFPC‐2 c) and P‐MFPC‐100 W d), water vapor adsorption isotherms of MFPC‐2 and P‐MFPCs at 25 °C e), the static atmospheric water adsorption profiles of MFPC‐2 and P‐MFPCs (5 mm packing thickness) at 25 °C and 30% RH f), the static and air‐blowing‐assisted dynamic atmospheric water adsorption profiles of P‐MFPC‐100 W with different packing thickness at 25 °C and 30% RH g), LMIH profiles of bare MFPC‐2 and P‐MFPCs (5 mm packing thickness) with 2.6 mT of magnetic field applied h), water desorption profiles of P‐MFPC‐100 W with different packing thickness driven by LMIH (2.6 mT of magnetic field) and traditional hot‐surface heating at 89 °C i), and LMIH‐driven water desorption profiles of P‐MFPC‐100 W (2 cm packing thickness) with different magnetic field applied j). Inset of i) shows the IR images of P‐MFPC‐100 W with LMIH (2.6 mT) for 8 min and hot‐surface heating at 89 °C for 20 min.

Considering that MFPC‐2 exhibited rapid atmospheric water adsorption and strong LMIH capability, the MFPC‐2 was selected to prepare P‐MFPCs through oxygen‐plasma treatments. Plasma treatment represents a facile and sustainable surface treatment technology that has been widely applied to functionalize the surfaces with abundant oxygen‐containing motifs.^[^ ^34^, ^35^ ^]^ After plasma treatment of the bare MFPC‐2 at 40, 70, and 100 W for 1.5 min (detailed in the experimental section of Supporting Information), a set of P‐MFPCs (respectively denoted as P‐MFPC‐40 W, P‐MFPC‐70 W, and P‐MFPC‐100 W) were prepared. Determined by the X‐ray photoelectron spectroscopy analysis (**Figure** **4**a), the relative atom ratios of O/C on the surface of P‐MFPC‐40 W, P‐MFPC‐70 W, and P‐MFPC‐100 W were 63.9%, 75.3%, and 82.3%, corresponding to 28.8%, 51.8%, and 65.9% enhancement than that of their parental MFPC‐2 (49.6%), respectively (Figure 4b). High‐resolution O 1s spectrum of the bare MFPC‐2 (Figure 4c) and P‐MFPC‐100 W (Figure 4d) showed three main peak components, assigned to carboxylic acid or ester (531.5 eV), carbonyl (ketone) (532.4 eV) and hydroxyl (533.9 eV).^35^ In comparison, the additionally introduced oxygen on the P‐MFPCs rendered by the plasma treatment mainly presented as hydroxyl and carboxylic acid or ester groups. Obviously, upon an oxygen plasma treatment, numerous additional oxygen‐containing moieties could be introduced onto the carbon surfaces. Moreover, such introduced oxygen content could be effectively regulated through the applied plasma energy.

Compared with the bare MFPC‐2, the P‐MFPC‐100 W showed similar surface morphologies, XRD patterns, and LMIH performance, albeit 7.6% of surface area loss due to the extra oxygen‐containing groups incorporated (Figure S3, Supporting Information). Even so, as determined by the water vapor adsorption isotherms at 25 °C, the P‐MFPCs exhibited greatly enhanced water adsorption capacities than the bare MFPC‐2 (Figure 3e). Of them, P‐MFPC‐100 W exhibited 0.12 g g^−1^ of water vapor adsorption capacity at 0.3 of *P/P_0_*, 41.2% higher than that of the bare MFPC‐2 (0.085 g g^−1^).

Toward the real‐world AWH application, the practical atmospheric water adsorption performance of the P‐MFPCs was firstly assessed by exposing the P‐MFPC powder (5 mm thickness) to a static air environment at 30% RH and 25 °C. As shown in Figure 3f, P‐MFPCs adsorbed 0.074–0.087 g g^−1^ of water in 30 min, 15.6%–35.9% higher than that of bare MFPC‐2 (0.064 g g^−1^). In comparison, P‐MFPC‐100 W reached a nearly water saturation in 30 min, while it took over 45 min for the bare MFPC‐2 to reach water saturation. Obviously, the oxygen plasma‐treatment could effectively improve the atmospheric water adsorption capability of the MFPCs at enhanced water adsorption kinetics. This was mainly attributed to the abundant hydrophilic oxygen‐containing motifs bearing on the P‐MFPC surfaces, which would greatly improve the P‐MFPC surface affinity to the water molecules and facilitate the atmospheric water adsorption. Under the same static atmospheric water adsorption measurement conditions, unfortunately, when the packing thickness of the applied P‐MFPC‐100 powder was increased to 1 cm and 2 cm, merely 0.075 and 0.035 g g^−1^ of water adsorption could be achieved although the water adsorption duration was extended to 2 h, respectively (Figure 4g). This suggested that increasing the thickness of the applied P‐MFPC‐100 powder resulted in the dramatic reduction in its atmospheric water adsorption kinetics, which was mainly caused by the insufficient surface exposure of the samples to the air under such static air conditions. This phenomenon was also commonly observed in the previously reported AWH sorbents,^[^ ^12^, ^15^, ^18^, ^21^ ^]^ representing one of the major challenges to hinder the scaled‐up application of the sorption‐based AWH.

To overcome this challenge, an air‐blowing‐assisted dynamic atmospheric water adsorption method was employed. For this purpose, a set‐up consisting of a sample tube (1.9 cm of inner diameter) with a filter paper cover on the top and an air inlet installed on the bottom was designed. In such a set‐up, forced by an airflow, the P‐MFPC powder could be easily blew up and violently flew around in the sample tube, thereby allowing the P‐MFPC powder to be rapidly mixed with and sufficiently exposed to the air. By means of such an air‐blowing‐assisted dynamic atmospheric water adsorption approach, it only took 10 min for the P‐MFPC‐100 W powder (5 mm packing thickness) to reach water saturation at 30% RH and 25 °C (Figure 4g), 3 times faster than the same measurement carried out at static air conditions (Figure 4f). More impressively, under the same air‐blowing‐assisted test conditions, further increase in the packing thickness of P‐MFPC‐100 W powder to 1 cm and 2 cm caused little compromise to its atmospheric water adsorption efficiency (Figure 4g). Therefore, distinct from the traditional static atmospheric water adsorption, air‐blowing‐assisted dynamic atmospheric water adsorption strategy could realize the highly efficient atmospheric water adsorption of the powder sorbent even when they were implemented at high packing thickness.

After being subjected to the static atmospheric water adsorption at 30% RH and 25 °C for 30 min, the LMIH‐driven water desorption performance of the P‐MFPCs were assessed by exposing them to a 2.6 mT of alternating magnetic field. As shown in Figure 4h, all the P‐MFPCs and the their parental MFPC‐2 exhibited over 91.3% and 100% of water desorption in 10 min and 20 min, respectively. More impressively, such highly efficient water desorption was also observed in the P‐MFPC‐100 W powder when it was applied in the 1 and 2 cm of packing thickness, corresponding to 95.6% and 95.3% of water desorption efficiency in 20 min, respectively (Figure 4i). As a comparison, a conventional hot‐surface heating‐driven water desorption process was also conducted by placing the water‐saturated P‐MFPC‐100 W (5 mm packing thickness) on a preheated hot‐surface at 89 °C that was similar to the LMIH with 2.6 mT of magnetic field applied for 8 min (inset of Figure 4i). It turned out that only 56.3% of water desorption efficiency could be achieved in 2.5 h (Figure 4i), significantly lower than the use of LMIH‐triggered water desorption strategy (100% of water desorption efficiency in 20 min, Figure 4j). Further investigations indicated that when the P‐MFPC‐100 W was applied in 2 cm packing thickness, lowering the applied magnetic field strength down to 1.5 mT merely resulted in 72.4% of water desorption efficiency even in 30 min. However, when the applied magnetic field strength was increased to 3.4 mT, a complete water desorption also could be rapidly achieved in 10 min.

Obviously, the use of LMIH could realize a dramatically enhanced water desorption efficiency than the traditional hot‐surface heating especially. Given the poor thermal conducting nature of the highly porous carbon materials and the very limited thermal contact between P‐MFPC powder and the hot‐plate, the use of traditional hot‐surface heating inevitably caused the uneven and insufficient heating of the P‐MFPC, resulting in the poor water desorption efficiency. Distinctly, since the CoNPs‐delivered LMIH homogeneously occurred within the P‐MFPCs, such generated heat was directly dissipated to and absorbed by its immediate carbon matrix, thereby leading to the rapid and uniform heating of the entire P‐MFPC. In addition to the excellent carbon material penetrating capacity of the magnetic field,^[^ ^26^ ^]^ the use of LMIH resulted in the outstanding water desorption efficiency of the P‐MFPCs even when they were utilized in high packing thickness.

**Figure 5:**
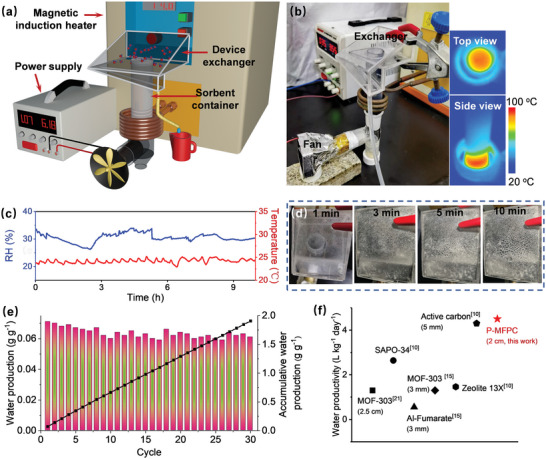
Schematic illustration a) and digital photograph b) of the presented atmospheric water harvester, the controlled air temperature and RH during the water production tests (c), inset of c) shows the IR images of the P‐MFPC‐100 W during the LMIH‐driven water desorption process, typical digital photographs of the evolved condensed water droplets on the exchanger wall of the prototype during its water‐desorption process d), water production of the prototype in each WHC e), and comparison of the practical water productivity at 30% RH and 25 °C between the previously reported state‐of‐the‐art AWH sorbents and our currently developed P‐MFPC‐100 W f).

As described above, synergistically benefiting from the large open microstructures, abundant surface oxygen‐containing groups, and feasibility for air‐blowing‐assisted dynamic atmospheric water adsorption, the P‐MFPCs exhibited extremely fast atmospheric water adsorption capacity (Figure 4g). Along with the highly efficient LMIH‐triggered water desorption capability (Figure 4j), P‐MFPCs held great promises for delivering fast‐cycling and high‐yield AWH. To validate this, a newly type of atmospheric water harvester consisting of a poly(methyl methacrylate) (PMMA) exchanger equipped with a magnetic induction heater, a forcing fan, and an air‐blowing‐assisted atmospheric water adsorption set‐up containing 3.2 g of MFPC‐100 W powder (2 cm packing thickness, **Figure** **5**a,b). The actual water production tests on the prototype were carried out in a controlled laboratory air environment. Through the simultaneously regulating the Constant Humidity Chamber and the laboratory air conditioner, the RH and temperature of the air environment were controlled to be 24–26 °C and 26–32% RH (Figure 5c). On the basis of the atmospheric water adsorption and water desorption profiles shown in Figure 4g,j, both the air‐blowing‐assisted atmospheric water capture phase and the LMIH‐triggered water desorption processes were respectively performed for 10 min. It thus would allow the prototype to take 20 min to complete a water harvesting cycle (WHC). The atmospheric water adsorption phase was initiated by switching on the forcing fan to allow the air to pass through the sample tube at 0.8 L min^−1^ of airflow rate. After such a dynamic atmospheric water capture process proceeded for 10 min, the forcing fan was switched off, allowing the water‐adsorbed sorbent powder to settle down on the bottom of the sample tube. Thereafter, a LMIH‐driven water desorption process was initiated by switching on the magnetic induction heater and proceeded for 10 min. To ensure the loaded sorbent with sufficient water desorption efficiency that was similar to what was described in Figure 4i (88.9 °C surface temperature), 3.4 mT of magnetic field was applied to the prototype (inset of Figure 5b). The released hot water vapor would be condensed into liquid water droplets on the inner walls of the prototype exchanger for collection (Figure 5d). To quantify the water productivity of the prototype, the produced water in each WHC was collected and measured by weight. Given that a WHC could be completed in 20 min, the continuous operation of the prototype for 9 h would allow 30 WHCs (Figure 5e). In each WHC, 0.060–0.068 g g^−1^ of water (0.063 g g^−1^ in average) was produced, corresponding to a 0.19 L_H2O_ kg^−1^ h^−1^ (3 WHCs) and 4.5 L_H2O_ kg^−1^ day^−1^ (72 WHCs) of water productivity. A further comparison indicated that no significant differences in the water production was observed between each WHC, evidencing the excellent reusability of the P‐MFPCs, which was further confirmed by that after 30 recycles the P‐MFPC‐100W showed the similar water vapor adsorption capacity to the freshly prepared P‐MFPC‐100W (Figure S4, Supporting Information). Determined by the inductively coupled plasma mass spectroscopy (ICP‐MS) characterizations, further investigations showed that the concentrations of the primary ions (K^+^, Ca^2+^, Na^+^, Mg^2+^, and Al^3+^) in the produced water were far below the World Health Organization standard (Figure S5, Supporting Information), indicating that the water produced with our currently presented prototype could meet the drinking needs.

To the best of our knowledge, our current developed P‐MFPC‐100 W exhibited among highest water productivity compared with the existing state‐of‐the‐art AWH sorbents at 30% RH (Figure 5f). This was mainly resulting from the ultra‐fast adsorption‐desorption cycling capacity of the P‐MFPC synergistically rendered by its flower‐like microstructure and abundant surface oxygen‐containing motifs, the use of the air‐blowing‐assisted dynamic atmospheric water adsorption method and the highly efficient LMIH‐driven water desorption strategy. More importantly, such an ultrafast water adsorption‐desorption cycling of the P‐MFPC was achieved when it was implemented as 2 cm of packing thickness, over 4 times thicker than the previously commonly utilized sorbents (<5 mm), evidencing that our currently presented P‐MFPCs hold great promise in scalable water production with a compact AWH devices.

## 3 Conclusion

The P‐MFPC was synthesized by the co‐carbonization of PAN and controlled content of ZIF‐67 in DES, followed by an oxygen plasma surface treatment. The resulting P‐MFPC exhibited flower‐like microstructures bearing abundant surface oxygen‐containing groups, which together with the use of an air‐blowing‐assisted dynamic water adsorption strategy enabled the P‐MFPC to demonstrate extremely fast atmospheric water adsorption kinetics. The P‐MFPC also showed outstanding LMIH capacity which could drive up to 100% of the adsorbed water in it to be highly efficiently released. Such highly efficient atmospheric water adsorption and LMIH‐driven water desorption capability of the P‐MFPCs synergistically enabled a P‐MFPC‐based AWH prototype to complete a WHC in 20 min and deliver a record 4.5 of water productivity at 30% RH and 25 °C even when 2 cm of packing thickness of the P‐MFPC was implemented, among the fastest water adsorption‐desorption cycling and the highest water production rate compared with the existing AWH devices where the applied packing thickness of the sorbents was commonly less than 5 mm. The synergistically enabling fast cycling and high‐yield AWH with our currently presented P‐MFPC, therefore, provides a promising strategy for the scalable AWH with the compact AWH devices.

## 4 Experimental Section

### Fabrication of Bare MFPC and P‐MFPCs

All reagents and solvents were commercially obtained from Aladdin (China) and used as received. ZIF‐67 was synthesized according to the previously reported procedures.^[^ ^36^ ^]^ MFPC‐2 was synthesized by co‐carbonization of PAN and ZIF‐67 based on the previously reported carbonization method.^[^ ^29^ ^]^ In brief, 0.75 of PAN and 0.4 g of ZIF67 were codispersed in a DES consisting of 2.1 g of urea and 0.68 g of ZnCl_2_ at 70 °C. The resulting reaction mixture was then transferred into a tubular high temperature furnace (KEJING, OTF‐1200X) and subjected to the calcination under nitrogen flow at 350 °C for 1 h and then 800 °C for 2 h. After repeated washing with deionized water for 24 h and drying under vacuum at 100 °C for 8 h, MFPC‐2 was synthesized. Following the similar protocols, regulating the ZIF‐67 using amount in the carbonization reaction systems to 0, 0.10 and 0.22 g resulted in the production of PC‐1, PC‐2, and MFPC‐1, respectively. To prepare P‐MFPCs, the bare MFPC‐2 powder was placed in the chamber of a plasma cleaner (Electronic Diener, ZEPTO) filled with oxygen atmosphere. After evacuating the chamber to low‐pressure residual oxygen (0.2 mbar). The MFPC‐2 was subjected to plasma treatment at 100 W for 30 s. Upon repeating such a treatment process for 3 times, P‐MFPC‐100 W was prepared. Following the similar processes, the use of 40 and 70 W of plasma power resulted in the preparation of P‐MFPC‐40 W and P‐MFPC‐70 W, respectively.

### Characterizations

The microstructures of samples were analyzed using a Hitachi S‐4800 SEM and JEOL‐1200 TEM. Powder X‐ray diffraction of samples was measured at Bruker D8 Advanced X‐ray Diffractometer operating under CuK*α* radiation (40 kV, 40 mA) equipped with a LynxEye detector. The diffraction patterns were collected in the 2*θ* range of 5–60° with a step size of 0.02° and a count time of 3.2 s step^−1^. Nitrogen adsorption and desorption isotherms were measured at ‐196 °C using a BELSORP‐max II automated gas sorption analyzer. The specific surface area was calculated by using Brunauer—Emmett–Teller (BET) method. Magnetic induction heating capacity of samples was carried out on a magnetic induction heater (SPZ‐45, Shenzhen Shuangping) under ambient conditions, followed by recording their surfaces temperature with a resolution of 0.1 °C using an infrared camera (Testo, 871) or a fluorescent thermometer (TMEAS, FM‐07).

Water vapor adsorption isotherms were recorded on a BELSORP‐max II automated gas sorption analyzer at 25 °C. The static atmospheric water capture experiments were conducted in a Constant Humidity Chamber (HWHS‐100L, Kaice Shanghai) with a controlled constant humidified air environment, and at 25 °C. The dynamic air‐blowing‐assisted atmospheric water adsorption tests were carried out at ambient laboratory conditions with a controlled temperature of ≈25 °C. The 30% RH of arid air was supplied by the Constant Humidity Chamber. LMIH‐triggered water desorption was performed on a magnetic induction heater (SPZ‐45, Shenzhen Shuangping) at ambient conditions (25 °C and 30% RH). All the water adsorption and desorption process were monitored by the mass change of samples.

## Conflict of Interest

The authors declare no conflict of interest.

## Supporting information

Supporting Information

## Data Availability

The data that support the findings of this study are available from the corresponding author upon reasonable request.
