# Supplementary material for: Synergistically Enabling Fast‐Cycling and High‐Yield Atmospheric Water Harvesting with Plasma‐Treated Magnetic Flower‐Like Porous Carbons
Source: Adv Sci (Weinh). 2022 Nov 24;10(3):2204840. doi: 10.1002/advs.202204840 (PMC9875688; doi:10.1002/advs.202204840)
Supplement: Supplementary file 1 — Supporting Information [file ADVS-10-2204840-s001.pdf]

ADVANCED SCIENCE
Open Access

Supporting Information

for *Adv. Sci.*, DOI 10.1002/advs.202204840

Synergistically Enabling Fast-Cycling and High-Yield Atmospheric Water Harvesting with Plasma-Treated Magnetic Flower-Like Porous Carbons

*Yifeng Ying, Guifang Yang, Yingle Tao, Qiannan Wu and Haiqing Li**

Synergistically Enabling Fast-Cycling and High-Yield Atmospheric Water Harvesting with Plasma-Treated Magnetic Flower-Like Porous Carbons

Yifeng Ying, Guifang Yang, Yingle Tao, Qiannan Wu, Haiqing Li*

State Key Laboratory of Materials-Oriented Chemical Engineering, College of Chemical Engineering, Nanjing Tech University, Nanjing 211816, China

E-mail: haiqing.li@njtech.edu.cn

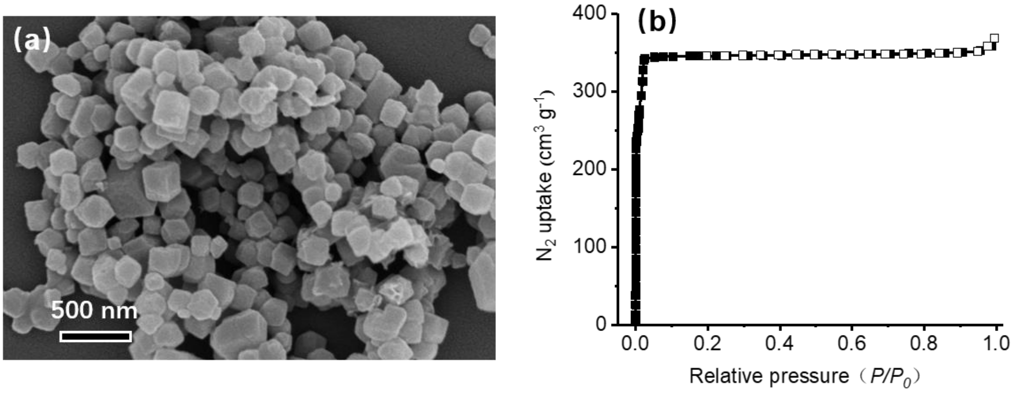

Figure S1. SEM images of bare ZIF-67 crystals (a) and its N_2 adsorption isotherm at -196 ^oC (b).

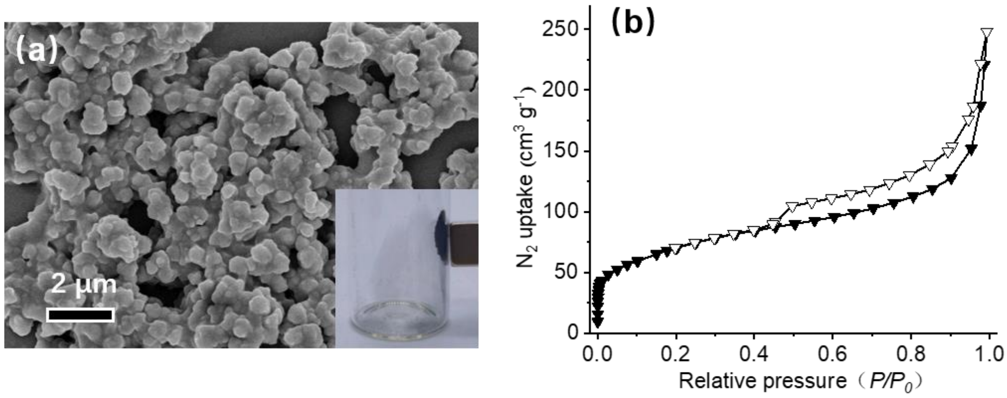

Figure S2. SEM image (a) and N_2 adsorption isotherm at -196 ^oC (b) of the magnetic porous carbon synthesized by using ZIF-67 alone as carbonaceous carbon precursor. Inset (a) shows the digital photograph of the corresponding porous carbon attracted with an external magnet.

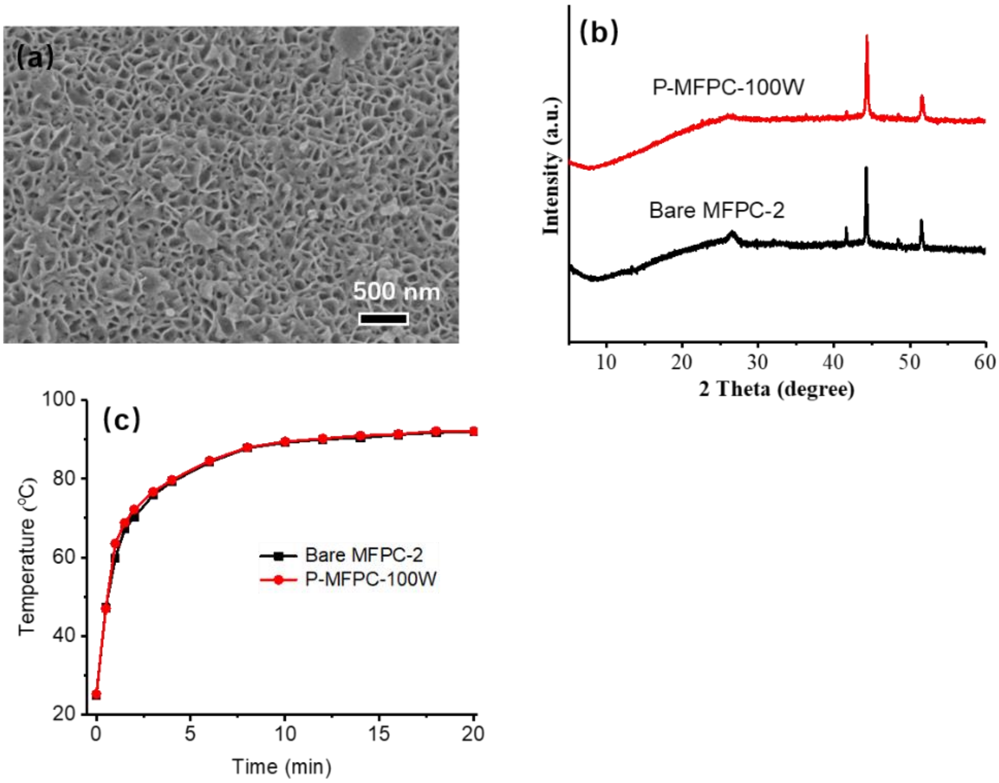

Figure S3. SEM image of P-MFPC-100W (a), XRD patterns (b) and atmospheric water adsorption (at 30% RH and 25 °C) profiles (b) of the P-MFPC-100W and its parental MFPC-2.

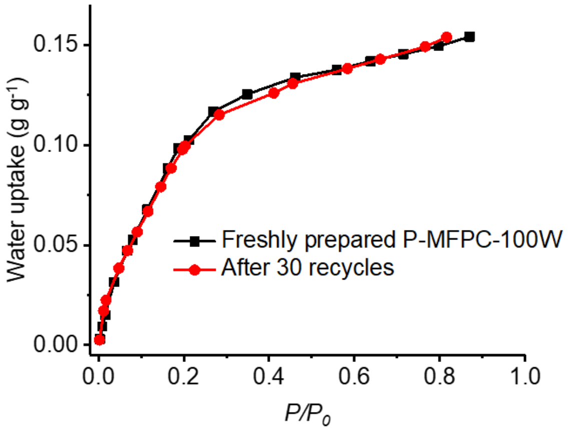

Figure S4. Water vapor adsorption isotherms of P-MFPC-100W at 25 °C before and after 30 reuses.

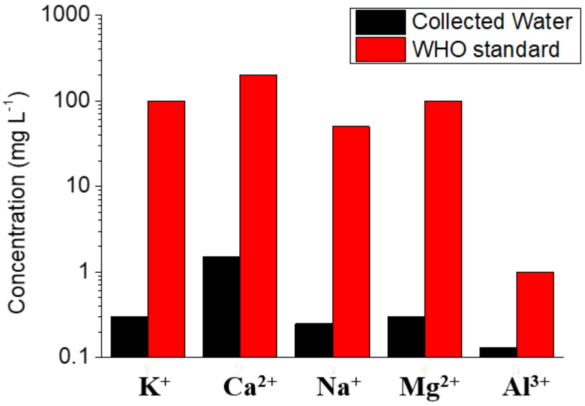

Figure S5. The quality of the produced water compared with the WHO standards.
